# Factors Influencing Neuromuscular Blockade Reversal Choice in the United States Before and During the COVID-19 Pandemic: Retrospective Longitudinal Analysis

**DOI:** 10.2196/52278

**Published:** 2024-07-22

**Authors:** Vladimir Turzhitsky, Lori D Bash, Richard D Urman, Michael Kattan, Ira Hofer

**Affiliations:** 1 Merck & Co, Inc Rahway, NJ United States; 2 College of Medicine The Ohio State University Columbus, OH United States; 3 Cleveland Clinic Cleveland, OH United States; 4 Icahn School of Medicine at Mount Sinai New York, NY United States

**Keywords:** neuromuscular blockade, sugammadex, neostigmine, rocuronium, vecuronium, intubation, counterfactual, anesthesia, anesthetic, anesthesiologist, anesthesiologists, surgery, surgical, preference, preferences, retrospective, utilization, pattern, patterns, trend, trends, national, healthcare database, healthcare databases, COVID-19, time-trend analysis, neuromuscular, longitudinal analysis, longitudinal, neuromuscular blockade agent, clinical, surgical procedure, inpatient, inpatient surgery, retrospective analysis, USA, United States

## Abstract

**Background:**

Neuromuscular blockade (NMB) agents are a critical component of balanced anesthesia. NMB reversal methods can include spontaneous reversal, sugammadex, or neostigmine and the choice of reversal strategy can depend on various factors. Unanticipated changes to clinical practice emerged due to the COVID-19 pandemic, and a better understanding of how NMB reversal trends were affected by the pandemic may help provide insight into how providers view the tradeoffs in the choice of NMB reversal agents.

**Objective:**

We aim to analyze NMB reversal agent use patterns for US adult inpatient surgeries before and after the COVID-19 outbreak to determine whether pandemic-related practice changes affected use trends.

**Methods:**

A retrospective longitudinal analysis of a large all-payer national electronic US health care database (PINC AI Healthcare Database) was conducted to identify the use patterns of NMB reversal during early, middle, and late COVID-19 (EC, MC, and LC, respectively) time periods. Factors associated with NMB reversal choices in inpatient surgeries were assessed before and after the COVID-19 pandemic reached the United States. Multivariate logistic regression assessed the impact of the pandemic on NMB reversal, accounting for patient, clinical, procedural, and site characteristics. A counterfactual framework was used to understand if patient characteristics affected how COVID-19–era patients would have been treated before the pandemic.

**Results:**

More than 3.2 million inpatients experiencing over 3.6 million surgical procedures across 931 sites that met all inclusion criteria were identified between March 1, 2017, and December 31, 2021. NMB reversal trends showed a steady increase in reversal with sugammadex over time, with the trend from January 2018 onwards being linear with time (*R*^2^>0.99). Multivariate analysis showed that the post–COVID-19 time periods had a small but statistically significant effect on the trend, as measured by the interaction terms of the COVID-19 time periods and the time trend in NMB reversal. A slight increase in the likelihood of sugammadex reversal was observed during EC relative to the pre–COVID-19 trend (odds ratio [OR] 1.008, 95% CI 1.003-1.014; *P*=.003), followed by negation of that increase during MC (OR 0.992, 95% CI 0.987-0.997; *P*<.001), and no significant interaction identified during LC (OR 1.001, 95% CI 0.996-1.005; *P*=.81). Conversely, active reversal (using either sugammadex or neostigmine) did not show a significant association relative to spontaneous reversal, or a change in trend, during EC or MC (*P*>.05), though a slight decrease in the active reversal trend was observed during LC (OR 0.987, 95% CI 0.983-0.992; *P*<.001).

**Conclusions:**

We observed a steady increase in NMB active reversal overall, and specifically with sugammadex compared to neostigmine, during periods before and after the COVID-19 outbreak. Small, transitory alterations in the NMB reversal trends were observed during the height of the COVID-19 pandemic, though these alterations were independent of the underlying NMB reversal time trends.

## Introduction

The neuromuscular blockade (NMB) agents rocuronium and vecuronium help achieve and maintain optimal levels of muscle paralysis to facilitate intubation and ensure patient immobility during surgery. Following surgery, recovery of neuromuscular function is accomplished via spontaneous recovery or through active pharmacologic reversal. Spontaneous recovery can be slow and unpredictable and can result in residual neuromuscular blockade (rNMB) associated with deleterious consequences, including muscle weakness, impaired respiration, and postoperative pulmonary complications [1-4]. The incidence of rNMB with spontaneous recovery can vary widely but can reach and exceed 50% [3,5-7].

Additionally, 2 pharmacologic agents, neostigmine and sugammadex, are available for active NMB reversal. Neostigmine is an anticholinesterase inhibitor, while sugammadex acts as a selective direct inhibitor of rocuronium and vecuronium that allows for rapid, predictable reversal, even at deep NMB levels. Following the approval of sugammadex in the United States in 2016, the proportion of procedures using active reversal (vs spontaneous reversal) steadily increased through mid-2019. This coincided with the growing use of sugammadex for reversal, though significant practice variability has been observed based on patient, procedural, and environmental factors [8,9].

At the start of the COVID-19 pandemic, hospitals rapidly adopted measures to reduce viral exposure and reallocated resources to emergency departments and intensive care units. For surgical units, elective procedures were largely postponed while recommendations favored anesthetic techniques aimed to minimize aerosolization and contamination of the environment [10-12]. For example, the use of rapid sequence intubation became common if not standard, and interventions to shorten postanesthesia care unit (PACU) stay duration, such as using efficient NMB reversal strategies, would be advantageous in minimizing exposure risk. However, initial studies during the early COVID-19 period had not revealed the long-lasting impacts of the pandemic on surgical practice [13-15]. A more in-depth assessment may reveal subtle changes in anesthesia practice as hospitals transitioned from early to late COVID-19 eras.

This study analyzes NMB reversal agent use patterns for US adult inpatient surgeries before and after the COVID-19 outbreak to determine whether pandemic-related practice changes affected use trends established before COVID-19. By understanding these trends, we can gain insight into how NMB management has evolved following COVID-19 and potentially recognize patient, procedural, and institutional factors that were associated with these changes. We hypothesize that the use of sugammadex for NMB reversal would accelerate in the post–COVID-19 period given the evidence demonstrating decreased PACU time and, potentially, diminished exposure to COVID-19 [16,17]. We make use of methods such as counterfactual analysis, which have been introduced as an effective approach for inferring causality on retrospective health care data in general [18-25] and impacts of the COVID-19 pandemic in particular [26,27].

## Methods

### Data Source

A retrospective analysis was conducted on US adult inpatient surgical procedures occurring between March 1, 2017, and December 31, 2021, within the PINC AI Healthcare Database (PHD) [28]. The PHD is a large, US hospital–based, service-level, all-payer database that contains information on inpatient discharges, primarily from geographically diverse nonprofit, nongovernmental, and community and teaching hospitals and health systems from rural and urban areas. Hospitals or health care systems submit administrative, health care use, and financial data from patient encounters. Inpatient admissions include over 108 million visits with more than 8 million per year since 2012, representing approximately 25% of annual US inpatient admissions.

### Ethical Considerations

This study used preexisting data with no identifiable information and therefore does not require institutional review board review per Federal Regulations for the Protection of Human Research Subjects (45CFR 46.102(e)) or patient consent [29]. All patient-related study data (eg, demographics, disease state, and information on billed services such as medications, laboratory tests, diagnostics, and therapeutic services) were accessed in compliance with the Health Insurance Portability and Accountability Act of 1996. This analysis was conducted and reported per the Strengthening the Reporting of Observational Studies in Epidemiology guidelines.

### Patient Selection

US adults aged ≥18 years and who received rocuronium or vecuronium during an inpatient surgical procedure were included. Exclusion criteria were a diagnosis of myasthenia gravis or renal failure, receiving pyridostigmine therapy, NMB reversal with both sugammadex and neostigmine, pregnancy (proxied by women undergoing obstetrical procedures), or those diagnosed with COVID-19 (for encounters occurring in 2020 and 2021). For any hospitalized patient undergoing multiple surgeries during a calendar 30-day period or a given inpatient stay, only the first surgery was included in the analysis.

For each eligible patient, information on demographics, clinical characteristics (eg, age, gender, anthropometrics, and comorbidities), insurance status, admission status (eg, elective, emergency, or trauma), site characteristics (eg, hospital size and geographic region), and anatomic location of the surgery were collected. Additionally, the type of NMB agent administered (rocuronium or vecuronium) as well as the reversal strategy (eg, neostigmine, sugammadex, or no active pharmacologic reversal) were recorded. The use of rocuronium or vecuronium for NMB was the inclusion criteria for this study due to the aim of quantifying NMB reversal practice changes in the sugammadex-eligible population. Data were categorized by time period in the following manner: baseline period (BP, March 1, 2017, to February 29, 2019); before COVID-19 era (BC, March 1, 2019, to February 29, 2020); early COVID-19 era (EC, April 1, 2020, to July 31, 2020); middle COVID-19 era (MC, August 1, 2020, to December 31, 2020); and late COVID-19 era (LC, January 1, 2021, to December 31, 2021). For this study, the post–COVID-19 period encompasses EC, MC, and LC time periods. The month of March 2020 was omitted in these analyses to account for a transition period and due to the unavailability of COVID-19 diagnostic information. The EC period was predominated by the early part of the breakout, the lack of information beyond testing for COVID-19 and implementing strict measures to reduce viral exposure within the hospital setting; the MC period correlated with the initial availability of COVID-19 vaccination for health care workers, thus (theoretically) lessening the impact of the pandemic on health care decisions; the LC period reflects when vaccines were available to the general public and restrictive infection control measures were loosened.

### Statistical Analyses

NMB use was summarized by characteristics using descriptive statistics. Similarly, NMB reversal strategies (ie, sugammadex, neostigmine, or no reversal) were summarized by time period, patient, site, and procedural characteristics using descriptive statistics.

### Multivariable Analysis

To identify factors related to NMB reversal choice during the COVID-19 and pre–COVID-19 eras, 2 multivariable logistic regression models were developed similarly to previous studies that modeled NMB reversal choices using PHD through June 2019 [8,9]. The first logistic regression models (model 1a and model 1b) aimed to test the effect of the COVID-19 time period on reversal choices by accounting for patient, hospital, and procedural characteristics. Encounters spanning both time periods (pre– and post–COVID-19) were included in these models to test for the overall effect of the COVID-19 era on reversal patterns, after accounting for all the covariates. Model 1a evaluated the effect of active (pharmacological) versus no (nonpharmacological) NMB reversal while model 1b evaluated sugammadex versus neostigmine reversal. Model 1 takes into account the trend in reversal over time by modeling the changes in NMB reversal as a linear trend over the period of January 1, 2018, to December 31, 2021. The effect of EC, MC, and LC eras are modeled as an interaction of the corresponding time period flag with the time-trend variable. For example, a positive interaction term coefficient between a post–COVID-19 period and time-trend variable in model 1b indicates a more rapidly increasing likelihood of sugammadex being used in the post–COVID-19 era as compared to the trend in the pre–COVID-19 time periods. The results from model 1 provide an overall estimate of the effect of the COVID-19 time period but do not provide insight into the effects for various population subtypes.

### Counterfactual Analysis

Model 2 was constructed and used in a counterfactual analysis to address model 1’s inability to evaluate changes in NMB reversal over time within patient subgroups. Models 2a (active vs no NMB reversal) and 2b (sugammadex vs neostigmine) were constructed using pre–COVID-19 data (January 1, 2018, to February 29, 2020) to be able to predict how a patient would have their NMB reversed (or not) based on their encounter characteristics. These models also include a continuous time variable that accounts for a linear trend to the log likelihood of the NMB reversal choice, to extrapolate this trend to the COVID-19 eras. Model accuracy, such as the receiver operating characteristic curve, is reported to help gauge the utility of these models for counterfactual analysis.

Counterfactual analysis was conducted to predict how COVID-19–era patients would have been reversed had they been treated during the pre–COVID-19 era based on their demographic, clinical, and institutional characteristics. The differences between the observed sugammadex reversal in the COVID-19 eras (actual) and the hypothetical or predicted reversal had each of those patients been seen pre–COVID-19 based on model 2 (counterfactual) were calculated. The differences in actual versus counterfactual reversal choices were then compared for each of the patient demographic, clinical, and institutional characteristics (eg, age group, comorbidities, surgery type, or hospital size). The counterfactual model was calibrated by adjusting the cutoff probability threshold to result in the same number of predicted classes (eg, sugammadex and neostigmine) as were actually observed in the combined COVID-19 eras. The odds ratios (OR) were also normalized such that the total patient-weighted OR was 1, which removed any residual time-dependent drift from the counterfactual model. The NMB reversal was compared between actual and counterfactual for each demographic, clinical, and institutional characteristic by obtaining ORs and CIs based on contingency tables for each covariate.

All statistical analyses were performed using SAS software (version 9.4; SAS Institute Inc).

## Results

### Study Population

Among the nearly 39.4 million inpatient encounters evaluated between March 1, 2017, and December 31, 2021, in the PHD, a total of 3,289,747 patients and 3,602,887 procedures involved the use of rocuronium or vecuronium and met all inclusion and exclusion criteria. The number of encounters included 1,644,370 during BP, 820,078 during BC, and 1,138,439 during the 3 post–COVID-19 periods (Table S1 in Multimedia Appendix 1). Patient demographics and characteristics were generally similar across the time periods despite attaining statistical significance driven by the large sample size (Table 1). Mean age (SD) ranged from 58.5 (16.76) years in the EC period to 59.0 (16.35) years during BC. A slightly higher percentage of patients were women (range 108,541/209,451, 51.8% in EC to 890,910/1,644,370, 54.2% in BP), and most patients identified as White (range 477,774/628,197, 76.1% in LC to 1,287,545/1,644,370, 78.3% in BP) throughout the study.

The percentage of patients with at least 1 comorbidity trended higher during this study’s period, increasing from 80.4% (1,321,911/1,644,370) during BP to 85.2% (535,076/628,197) by LC. The largest increases in comorbidity rates (>2% increase from BP to LC) were observed in cardiac arrhythmias, fluid or electrolyte disorders, and obesity or overweight conditions. The percentage of admissions due to elective procedures decreased between BC and EC (from 451,190/820,078, 55%, to 98,637/209,451, 47.1%) and there was a corresponding rise in the percentage of emergency or urgent admissions during these time periods (from 348,840/820,078, 42.5%, during BC to 104,123/209,451, 49.7% during EC).

Among the 3.6 million patient encounters included in this analysis, a majority involved teaching hospitals (range 885,068/1,644,370, 53.8%, in BP to 358,262/628,197, 57% in LC) and approximately 90% occurred in urban institutions (range 188,028/209,451, 89.8% in EC to 571,412/828,197, 91% in LC, Table S2 in Multimedia Appendix 1). The largest proportion of encounters (1,516,497/3,602,887, 42.1%) involved hospitals with 500 or more beds, while institutions with fewer than 200 beds accounted for approximately 15.5% (559,884/3,602,887) of encounters. Nearly half (1,736,173/3,602,887, 48.2%) of the encounters involved institutions in the South, 23% (828,275/3,602,887) from the Midwest, 14.6% (525,401/3,602,887) from the West, and the remaining 14.2% (513,038/3,602,887) from the Northeast.

**Table 1:** Patient characteristics.

		BP^a^ (n=1,644,370)		BC^b^ (n=820,078)		EC^c^ (n=209,451)		MC^d^ (n=300,791)		LC^e^ (n=628,197)
**Age^f^ (years)**										
	Mean (SD)	58.6 (16.31)	59.0 (16.35)		58.5 (16.76)		58.6 (16.57)		58.8 (16.85)	
	Minimum, Maximum	18, 89	18, 89		18, 89		18, 89		18, 89	
	Median (IQR)	61 (48-70)	61 (48-71)		61 (47-71)		61 (48-71)		61 (47-71)	
**Age category^f^ (years), n (%)**										
	18-30	112,133 (6.8)	54,225 (6.6)		15,683 (7.5)		21,128 (7)		45,338 (7.2)	
	31-40	147,963 (9)	73,238 (8.9)		19,839 (9.5)		28,467 (9.5)		61,031 (9.7)	
	41-50	217,072 (13.2)	105,670 (12.9)		27,043 (12.9)		39,092 (13)		80,868 (12.9)	
	51-60	340,761 (20.7)	162,767 (19.8)		40,873 (19.5)		58,449 (19.4)		117,202 (18.7)	
	61-70	416,743 (25.3)	207,379 (25.3)		51,304 (24.5)		74,935 (24.9)		153,046 (24.4)	
	71-80	288,194 (17.5)	153,377 (18.7)		38,255 (18.3)		55,684 (18.5)		118,827 (18.9)	
	>80	121,504 (7.4)	63,422 (7.7)		16,454 (7.9)		23,036 (7.7)		51,885 (8.3)	
Sex female^f^, n (%)		890,910 (54.2)		440,317 (53.7)		108,541 (51.8)		159,893 (53.2)		333,931 (53.2)
**Race^f^, n (%)**										
	Asian	25,205 (1.5)	13,155 (1.6)		3811 (1.8)		5502 (1.8)		13,947 (2.2)	
	Black	172,317 (10.5)	86,619 (10.6)		22,820 (10.9)		34,421 (11.4)		74,791 (11.9)	
	White	1,287,545 (78.3)	634,056 (77.3)		163,018 (77.8)		232,506 (77.3)		477,774 (76.1)	
Hispanic ethnicity^f^, n (%)		141,183 (8.6)		73,837 (9)		18,426 (8.8)		27,853 (9.3)		68,885 (11)
**Insurance^f,g^, n (%)**										
	Commercial	665,441 (40.5)	317,972 (38.8)		79,909 (38.2)		115,013 (38.2)		231,540 (36.9)	
	Government	749,389 (45.6)	383,018 (46.7)		96,045 (45.9)		138,052 (45.9)		289,800 (46.1)	
	Low-income	212,173 (12.9)	108,509 (13.2)		30,647 (14.6)		43,737 (14.5)		97,210 (15.5)	
Comorbidites ≥1^f^, n (%)		1,321,911 (80.4)		676,491 (82.5)		175,939 (84)		252,355 (83.9)		535,076 (85.2)
**Comorbidities^h^, n (%)**										
	Cardiac arrhythmias^f^	278,499 (16.9)	148,374 (18.1)		39,801 (19)		56,421 (18.8)		124,456 (19.8)	
	Chronic pulmonary disease^f^	303,871 (18.5)	157,402 (19.2)		40,808 (19.5)		58,895 (19.6)		123,865 (19.7)	
	Congestive heart failure^f^	114,852 (7)	64,837 (7.9)		18,118 (8.7)		25,313 (8.4)		57,681 (9.2)	
	Depression^f^	216,235 (13.2)	115,225 (14.1)		29,414 (14)		44,250 (14.7)		91,828 (14.6)	
	Diabetes (complicated)^f^	131,594 (8)	74,660 (9.1)		21,230 (10.1)		29,410 (9.8)		65,836 (10.5)	
	Diabetes (uncomplicated)^f^	223,314 (13.6)	108,624 (13.2)		26,276 (12.5)		38,592 (12.8)		80,350 (12.8)	
	Fluid or electrolyte disorders^f^	325,819 (19.8)	174,481 (21.3)		52,486 (25.1)		70,018 (23.3)		152,579 (24.3)	
	Hypothyroidism^f^	200,129 (12.2)	103,612 (12.6)		25,704 (12.3)		37,537 (12.5)		78,411 (12.5)	
	Obesity or overweight^f^	394,421 (24)	210,688 (25.7)		54,282 (25.9)		82,251 (27.3)		173,667 (27.6)	
	Other neurological disorders^f^	117,534 (7.1)	63,352 (7.7)		18,501 (8.8)		25,511 (8.5)		55,371 (8.8)	
	Peripheral vascular disorders^f^	125,078 (7.6)	67,508 (8.2)		18,339 (8.8)		26,595 (8.8)		58,697 (9.3)	
	Sleep apnea^f^	147,851 (9)	81,705 (10)		19,498 (9.3)		30,259 (10.1)		65,179 (10.4)	
	Solid tumor without metastasis^f^	194,163 (11.8)	98,675 (12)		27,302 (13)		36,644 (12.2)		81,145 (12.9)	
COVID-19 not present^f,i^, n (%)		1,644,142 (100)		819,852 (100)		209,200 (99.9)		299,296 (99.5)		601,470 (95.7)
**Admission type^f^, n (%)**										
	Elective	932,608 (56.7)	451,190 (55)		98,637 (47.1)		156,479 (52)		307,003 (48.9)	
	Emergency or urgent	674,323 (41)	348,840 (42.5)		104,123 (49.7)		136,181 (45.3)		302,208 (48.1)	
	Trauma center	37,439 (2.3)	20,048 (2.4)		6691 (3.2)		8131 (2.7)		18,986 (3)	

^a^BP: baseline period.

^b^BC: before COVID-19 era.

^c^EC: early COVID-19 era.

^d^MC: middle COVID-19 era.

^e^LC: late COVID-19 era.

^f^Statistically significant at the *P*<.05 level.

^g^Commercial category includes managed care, workers’ compensation, and self-pay. The government category includes Medicare and other government insurance types. The low-income category includes Medicaid, charity, and indigent.

^h^Most frequently observed Elixhauser comorbidities shown.

^i^No history of COVID-19 within 2 months of encounter.

### NMB Use Patterns

During the total study period, the vast majority of encounters involved rocuronium use with or without succinylcholine (3,229,651 encounters, 89.6%; Table S3 in Multimedia Appendix 1). A general trend of increasing rates of rocuronium only (with or without succinylcholine) was observed during this study’s period, increasing from 87.1% during BP to 93% during LC. The use of succinylcholine with rocuronium or vecuronium was used in 5.3% of patient encounters overall. This rate increased from 4.8% during BP to a peak of 6.9% during the EC period, before falling to 5.4% during LC.

### NMB Reversal Agent Use Patterns

**Figure 1:**
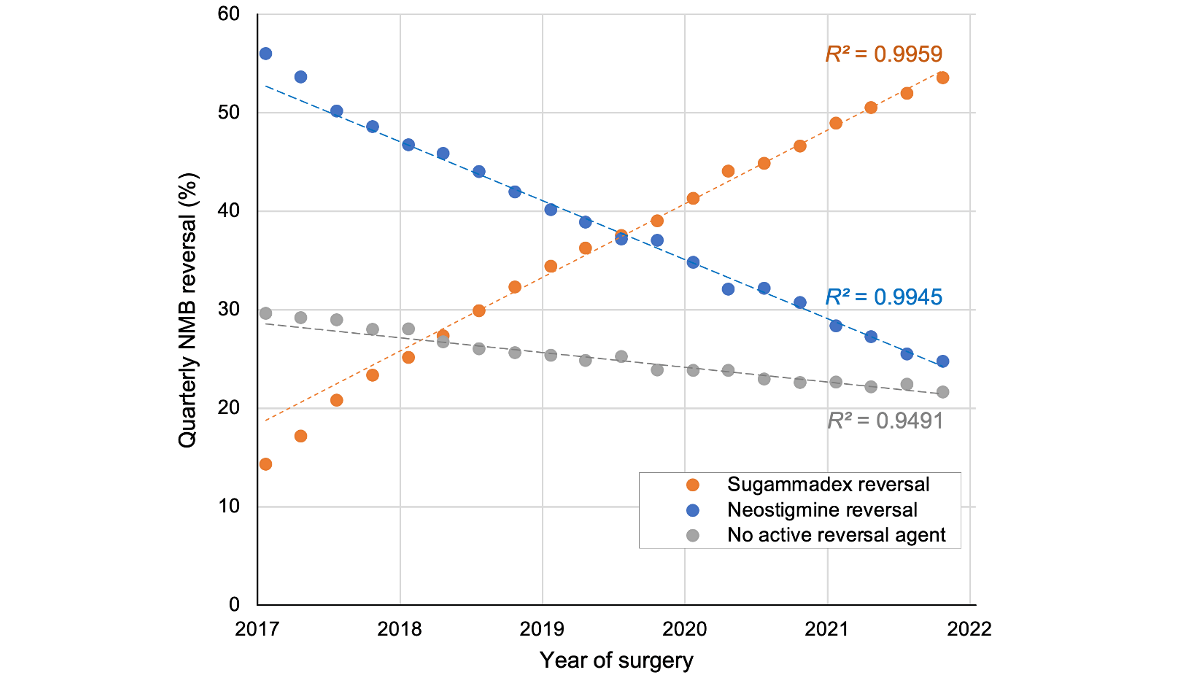
Quarterly proportions of NMB reversal agent use during this study’s period. Trend lines evaluate linearity after January 2018, the time period used for logistic regression models. NMB: neuromuscular blockade.

Before the COVID-19 outbreak, the use of sugammadex for NMB reversal steadily increased following its approval in 2016, with approximately 1 in 4 encounters using this agent for reversal during BP (Table 2; Figure 1) [9]. This trend continued through the post–COVID-19 eras, reaching 51.1% (321,268/628,197) of encounters during LC. Consequently, reversal with neostigmine decreased from 47.1% from BP to 26.6% during LC. Overall, the rate of active NMB reversal with either sugammadex or neostigmine gradually increased over time. Spontaneous reversal steadily decreased from 27.5% (451,838/1,644,370) of encounters during BP to 22.3% (139,854/628,197) during LC. The trends in sugammadex, neostigmine, and active reversal were approximately linear from 2018 until the end of this study’s period (*R*^2^>0.99, for sugammadex and neostigmine, *R*^2^=0.95 for active reversal).

When comparing patient characteristics by reversal type (ie, spontaneous, sugammadex, or neostigmine), the distribution by age, race, and ethnicity was similar, though statistical significance was achieved due to the large sample size (Tables S4-S6 in Multimedia Appendix 1). Encounters involving reversal with neostigmine or sugammadex tended to involve younger patients (mean 57.4, SD 17.15 to 58.2, SD 16.56 years for neostigmine and 58.7, SD 16.83 to 59.2, SD 16.43 years for sugammadex) compared to spontaneous reversal (mean 59.2, SD 16.28 to 59.9, SD 15.83 years). Women comprised a higher proportion of those reversed with sugammadex (49,534/92,709, 53.4%, to 231,852/417,266, 55.6%) or neostigmine (36,704/67,321, 54.5%, to 441,284/775,266, 56.9%) and a lower proportion of spontaneous reversal (22,303/49,421, 45.1%, to 217,774/451,838, 48.2%) compared to men. Those who underwent spontaneous reversal were more likely to have ≥1 comorbidity (379,136/451,838, 83.9% to 124,367/139,854, 88.9%) compared to those reversed with sugammadex (334,071/417,266, 80.1%, to 273,297/321,268, 85.1%) or neostigmine (608,704/775,266, 78.5%, to 137,412/167,075, 82.2%).

The use of NMB reversal agents was similar based on institution type. During BP, sugammadex was used in 24.8% (219,463/885,068) of encounters in teaching hospitals and 26.1% (197,803/759,302) in nonteaching hospitals. The use of sugammadex increased to 50.2% (179,721/358,262) among teaching hospitals and 52.4% (141,547/269,935) in nonteaching hospitals during the LC era.

**Table 2:** Pharmacological and nonpharmacological reversal of NMB^a^ during COVID-19 time periods.

Reversal strategy	Total (n=3,602,887)	Baseline period (n=1,644,370)	Before COVID (n=820,078)	Early COVID (n=209,721)	Middle COVID (n=300,791)	Late COVID (n=628,197)
Neostigmine, n (%)	1,411,570 (39.2)	775,266 (47.1)	307,727 (37.5)	67,321 (32.1)	94,181 (31.3)	167,075 (26.6)
Sugammadex, n (%)	1,280,618 (35.5)	417,266 (25.4)	311,227 (38)	92,709 (44.3)	138,148 (45.9)	321,268 (51.1)
No active reversal, n (%)	910,699 (25.3)	451,838 (27.5)	201,124 (24.5)	49,421 (23.6)	68,462 (22.8)	139,854 (22.3)

^a^NMB: neuromuscular blockade.

### Multivariable Analysis

**Figure 2:**
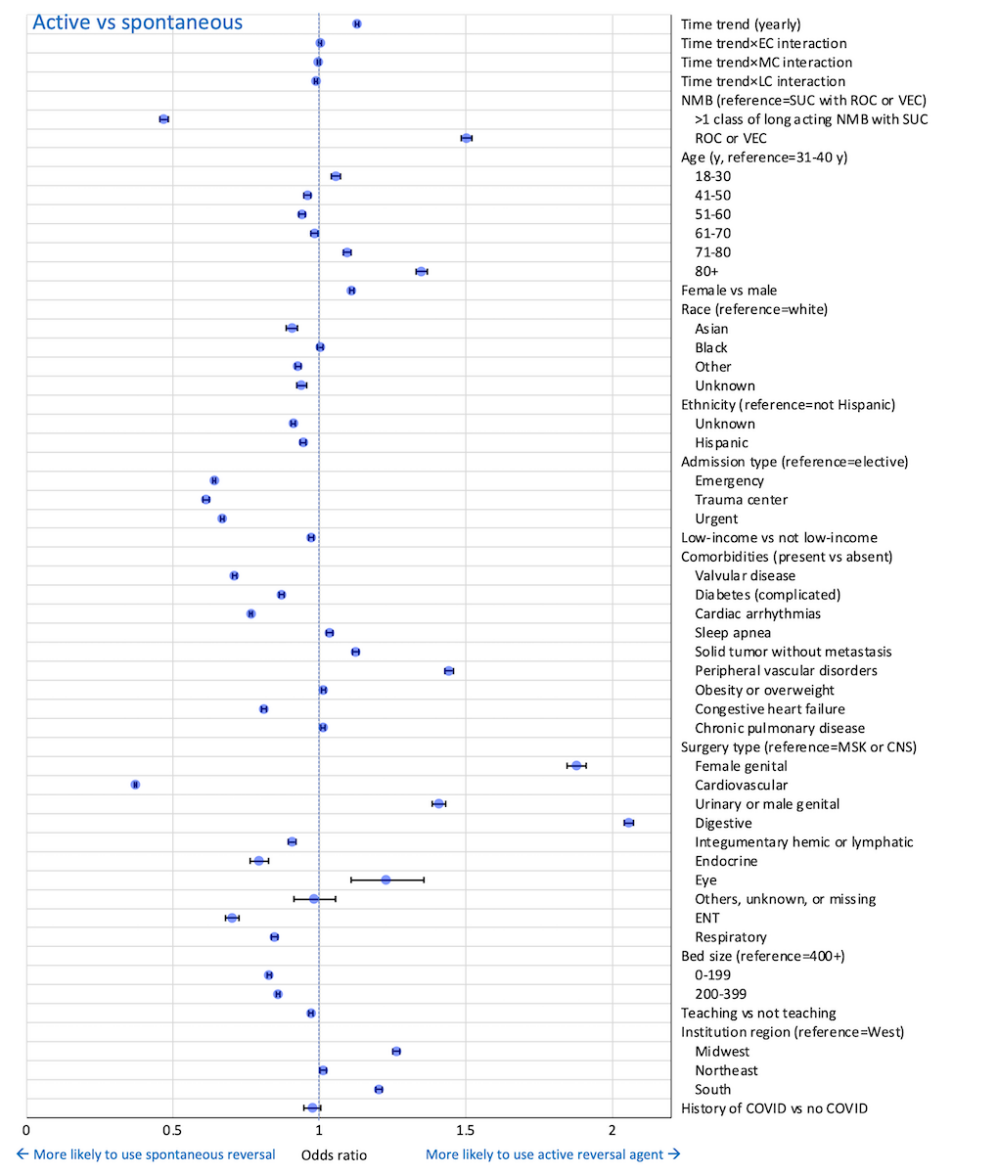
Forest plot of odds ratio: active versus spontaneous reversal (model 1a). The time period between January 1, 2018, and February 29, 2020, was considered as a reference to evaluate the interaction of the time trend with EC, MC, and LC periods. Bars represent 95% CI. Low income includes Medicaid, charity, and indigent insurance types. CNS: central nervous system; EC: early COVID-19 (April 1, 2020, and July 31, 2020; the month of March 2020 was omitted to account for a transition period and due to the unavailability of COVID-19 diagnostic information); ENT: ear nose throat; LC: late COVID-19 (January 1, 2021, to December 31, 2021); MC: middle COVID-19 (August 1, 2020, to December 31, 2020); MSK: musculoskeletal; NMB: neuromuscular blockade; ROC: rocuronium; SUC: succinylcholine; VEC: vecuronium.

**Figure 3:**
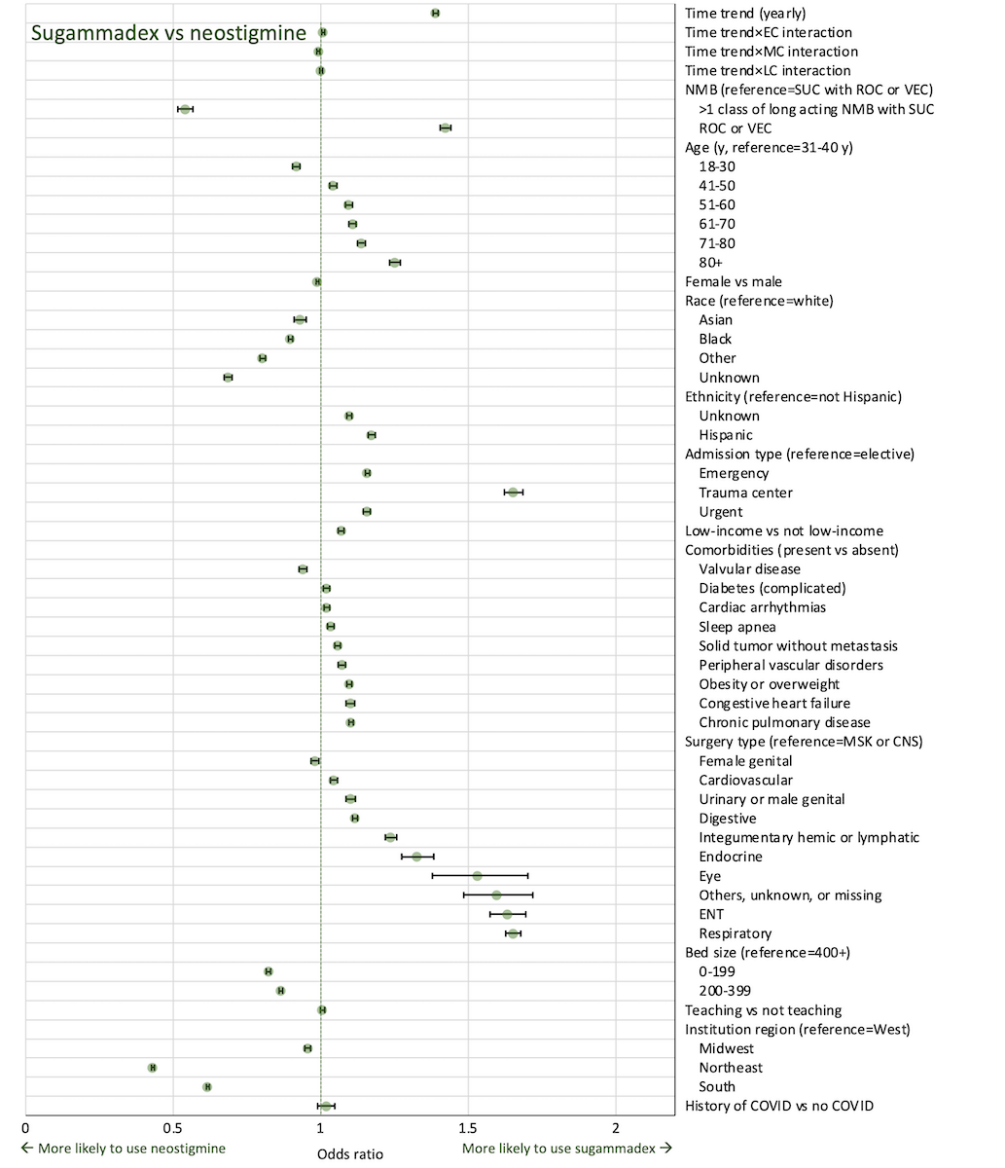
Forest plot of odds ratio: sugammadex vs neostigmine (model 1b). The time period between January 1, 2018, and February 29, 2020, was considered as a reference to evaluate the interaction of the time trend with EC, MC, and LC periods. Bars represent 95% CI. Low income includes Medicaid, charity, and indigent insurance types. CNS: central nervous system; EC: early COVID-19 (April 1, 2020, to July 31, 2020; the month of March 2020 was omitted to account for a transition period and due to the unavailability of COVID-19 diagnostic information); ENT: ear nose throat; LC: late COVID-19 (January 1, 2021, to December 31, 2021); MC: middle COVID-19 (August 1, 2020, to December 31, 2020); MSK: musculoskeletal; NMB: neuromuscular blockade; ROC: rocuronium; SUC: succinylcholine; VEC: vecuronium.

Multivariable regression analyses were used to identify time trends and their interaction terms with post–COVID-19 time periods for pharmacologic (active) versus no pharmacologic (spontaneous) reversal (model 1a), and for reversal with sugammadex versus neostigmine (model 1b; Table 3; Figures 2 and 3). The overall yearly time-trend throughout this study’s period demonstrated an increase in the use of active reversal (using either sugammadex or neostigmine) compared to no pharmacologic reversal (OR 1.129; *P*<.001). However, there were variations in the interaction term coefficient when analyzing specific post–COVID-19 time periods (refer to the Multivariable Analysis section of the Methods section for details on the analysis approach). Active reversal did not show a significant association, or change in trend, during EC (OR 1.002, 95% CI 0.997-1.008; *P*=.44) or MC (OR 0.996, 95% CI 0.991-1.001; *P*=.12). A slight but statistically significant decrease in the active reversal trend (ie, there was a slowing of the trend toward increased use of active reversal) was observed in LC (OR 0.987, 95% CI 0.983-0.992; *P*<.001). Based on these observations, the counterfactual analysis (model 2a) was not evaluated.

Significant associations in the use of active reversal were also observed based on patient, procedure, and institutional factors (Table 3; Figure 2). Except for those aged 18-30 years, fewer older adults (aged 41 to 70 years) tended to show a decreased odds of active reversal (OR range 0.941-0.983; reference those aged 31-40 years), while older adults (aged >70 years) were more likely to use active reversal (OR range 1.094-1.349). Compared to elective surgical procedures (reference), emergency, trauma, and urgent admissions revealed significantly decreased use of active pharmacologic reversal (OR 0.641, 95% CI 0.637-0.645; 0.612, 95% CI 0.602-0.622; and 0.668, 95% CI 0.662-0.675; respectively; *P*<.001 for each).

When comparing the use of sugammadex versus neostigmine in model 1b (Table 3, Figure 3), the yearly time-trend from January 2018 onwards demonstrated a steady increase in the use of sugammadex over neostigmine (OR 1.388, 95% CI 1.381-1.396; *P*<.001). Analysis of the specific post–COVID-19 time periods revealed a small but statistically significant interaction with the time trend in NMB reversal (Table 3). A slight but statistically significant increase in sugammadex reversal was observed during EC (OR 1.008, 95% CI 1.003-1.014; *P*=.003), followed by negation of that trend during MC (OR 0.992, 95% CI 0.987-0.997; *P*<.001). There was no significant interaction identified in the LC period (OR 1.001, 95% CI 0.996-1.005; *P*=.81).

Other covariates in model 1b that were significantly associated with sugammadex reversal included older age categories, urgent or emergent and trauma admissions, cardiac comorbidities (including arrhythmias, peripheral vascular disorders, congestive heart failure), obesity, chronic pulmonary disease, and diabetes. Most surgical types were associated with higher rates of sugammadex reversal as compared to the reference (musculoskeletal surgeries or surgeries involving the nervous system) with the exception of female genitalia. Hospitals with fewer beds (0-199 or 200-399 vs 400+) were associated with a lower likelihood of sugammadex reversal. Hospitals in the Northeast and South geographic regions of the United States also had significantly lower odds of sugammadex reversal as compared to the West and Midwest.

**Table 3:** Logistic regression estimates from multivariate models (active vs spontaneous and sugammadex vs neostigmine).

		Model 1a: active vs spontaneous		Model 1b: sugammadex vs neostigmine	
		Odds ratio (95% CI)	*P* value	Odds ratio (95% CI)	*P* value
Time trend (yearly)		1.129 (1.123-1.135)	<.001	1.388 (1.381-1.396)	<.001
Time trend×EC^a^		1.002 (0.997-1.008)	.44	1.008 (1.003-1.014)	.003
Time trend×MC^b^		0.996 (0.991-1.001)	.13	0.992 (0.987-0.997)	<.001
Time trend×LC^c^		0.987 (0.983-0.992)	<.001	1.001 (0.996-1.005)	.81
**NMB group (reference=rocuronium+succinylcholine or vecuronium+succinylcholine)**					
	>1 class of long-acting NMB + succinylcholine	0.469 (0.455-0.483)	<.001	0.542 (0.517-0.567)	<.001
	Rocuronium or vecuronium	1.501 (1.484-1.519)	<.001	1.421 (1.404-1.440)	<.001
**Age (y; reference=31-40 y)**					
	18-30	1.057 (1.041-1.072)	<.001	0.917 (0.904-0.930)	<.001
	41-50	0.959 (0.947-0.972)	<.001	1.042 (1.030-1.054)	<.001
	51-60	0.941 (0.930-0.952)	<.001	1.095 (1.083-1.107)	<.001
	61-70	0.983 (0.971-0.995)	.004	1.108 (1.095-1.120)	<.001
	71-80	1.094 (1.080-1.108)	<.001	1.137 (1.123-1.150)	<.001
	>80	1.349 (1.329-1.369)	<.001	1.251 (1.233-1.269)	<.001
Female vs male		1.110 (1.103-1.116)	<.001	0.989 (0.983-0.995)	<.001
**Race (reference=White)**					
	Asian	0.905 (0.887-0.925)	<.001	0.929 (0.909-0.950)	<.001
	Black	1.002 (0.993-1.012)	.62	0.897 (0.889-0.905)	<.001
	Other	0.926 (0.916-0.937)	<.001	0.803 (0.794-0.812)	<.001
	Unknown	0.938 (0.921-0.956)	<.001	0.686 (0.673-0.698)	<.001
**Ethnicity (reference=not Hispanic)**					
	Hispanic	0.945 (0.935-0.955)	<.001	1.171 (1.159-1.184)	<.001
	Unknown	0.910 (0.903-0.918)	<.001	1.096 (1.087-1.105)	<.001
**Admission type (reference=elective)**					
	Emergency	0.641 (0.637-0.645)	<.001	1.158 (1.150-1.165)	<.001
	Trauma center	0.612 (0.602-0.622)	<.001	1.652 (1.621-1.685)	<.001
	Urgent	0.668 (0.662-0.675)	<.001	1.156 (1.145-1.168)	<.001
Low-income (reference=not low-income^d^)		0.972 (0.964-0.981)	<.001	1.069 (1.060-1.079)	<.001
**Comorbidities (present vs absent)**					
	Valvular disease	0.710 (0.702-0.718)	<.001	0.940 (0.927-0.953)	<.001
	Diabetes (complicated)	0.870 (0.862-0.878)	<.001	1.019 (1.009-1.030)	<.001
	Cardiac arrhythmias	0.766 (0.760-0.772)	<.001	1.020 (1.012-1.029)	<.001
	Sleep apnea	1.034 (1.024-1.044)	<.001	1.034 (1.024-1.044)	<.001
	Solid tumor without metastasis	1.124 (1.113-1.135)	<.001	1.057 (1.047-1.067)	<.001
	Peripheral vascular disorders	1.443 (1.428-1.457)	<.001	1.071 (1.059-1.084)	<.001
	Obesity or overweight	1.013 (1.006-1.020)	<.001	1.096 (1.089-1.104)	<.001
	Congestive heart failure	0.810 (0.802-0.819)	<.001	1.100 (1.087-1.114)	<.001
	Chronic pulmonary disease	1.012 (1.004-1.019)	.002	1.102 (1.094-1.110)	<.001
**Surgical type (reference=MSK^e^ and CNS^f^)**					
	Female genital	1.877 (1.845-1.911)	<.001	0.980 (0.967-0.994)	.005
	Cardiovascular	0.372 (0.369-0.376)	<.001	1.044 (1.033-1.055)	<.001
	Urinary and male genital	1.408 (1.386-1.430)	<.001	1.101 (1.085-1.116)	<.001
	Digestive	2.056 (2.040-2.072)	<.001	1.115 (1.107-1.123)	<.001
	Integumentary hemic and lymphatic	0.906 (0.893-0.919)	<.001	1.237 (1.218-1.256)	<.001
	Endocrine	0.794 (0.764-0.825)	<.001	1.326 (1.274-1.381)	<.001
	Eye	1.226 (1.108-1.356)	<.001	1.531 (1.377-1.702)	<.001
	Others, unknown, or missing	0.981 (0.912-1.056)	.614	1.596 (1.484-1.717)	<.001
	ENT^g^	0.702 (0.680-0.724)	<.001	1.632 (1.572-1.695)	<.001
	Respiratory	0.847 (0.835-0.858)	<.001	1.651 (1.627-1.676)	<.001
**Bed size (reference=400+)**					
	0-199	0.828 (0.820-0.835)	<.001	0.823 (0.815-0.830)	<.001
	200-399	0.858 (0.852-0.865)	<.001	0.864 (0.858-0.870)	<.001
Teaching vs not teaching		0.971 (0.964-0.977)	<.001	1.006 (0.999-1.012)	.09
**Institution region (reference=West)**					
	Midwest	1.263 (1.250-1.275)	<.001	0.956 (0.947-0.966)	<.001
	Northeast	1.013 (1.002-1.024)	.021	0.431 (0.426-0.435)	<.001
	South	1.203 (1.192-1.213)	<.001	0.617 (0.612-0.623)	<.001
History of COVID-19 (reference=no COVID-19)		0.976 (0.947-1.005)	.10	1.018 (0.989-1.048)	.23

^a^EC: early COVID-19 era.

^b^MC: middle COVID-19 era.

^c^LC: late COVID-19 era.

^d^Low-income insurance types include Medicaid, charity, and indigent.

^e^MSK: musculoskeletal.

^f^CNS: central nervous system.

^g^ENT: ear nose throat.

### Counterfactual Analysis

**Figure 4:**
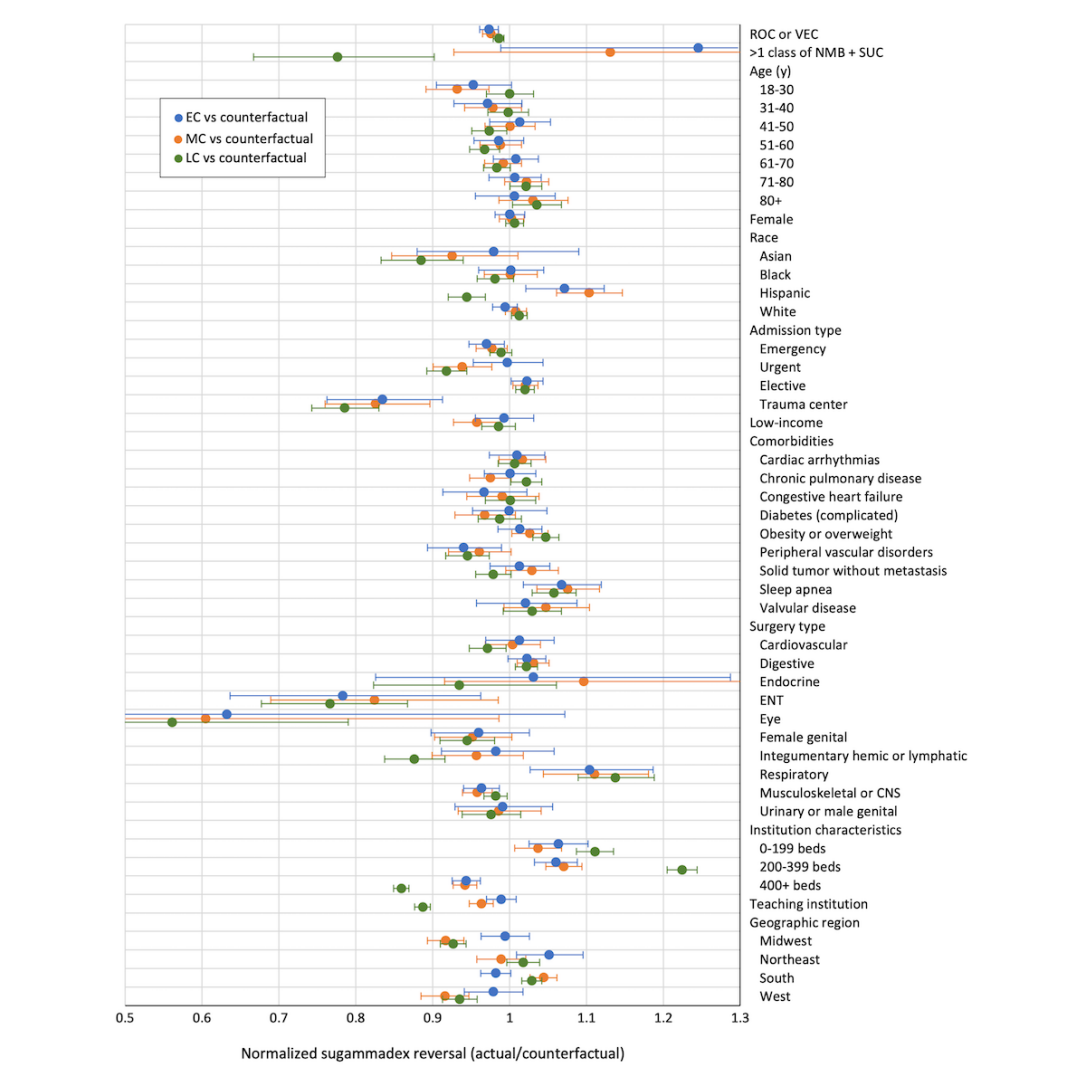
Counterfactual analysis comparing actual sugammadex reversal odds relative to prepandemic multivariate model–based reversal odds (model 2b). Normalized ORs were calculated by dividing observed ORs with counterfactual ORs of sugammadex reversal and multiplied by a frequency-weighted scaling factor for relative comparison between time periods. Bars represent 95% CI. Low income includes Medicaid, charity, and indigent insurance types. CNS: central nervous system; ENT: ear nose throat; MSK: musculoskeletal; NMB: neuromuscular blockade; OR: odds ratio; ROC: rocuronium; SUC: succinylcholine; VEC: vecuronium.

When comparing EC, MC, and LC time periods within patient subgroups, only a few differences were observed in actual NMB reversal compared to expected use. Most differences were observed in LC among institution and multimodal NMB characteristics (Figure 4). After normalization, only a few patient and institutional characteristics showed a significant deviation from the expected trend of the sugammadex reversal rate. Of the patient characteristics that had an observable counterfactual difference, patients with Hispanic ethnicity were reversed with sugammadex less frequently in the LC era as compared to how they would have been reversed before COVID-19. Institutions with 400 or more beds or classified as teaching institutions also had a relative decrease in sugammadex reversal rates as compared to expected trends from before COVID-19 data. On the other hand, small hospitals (0-199 beds), mid-sized hospitals (200-399), and those located in the south of the United States had higher rates of sugammadex reversal than expected from pre–COVID-19 trends, having relative actual or counterfactual ratios greater than 1.

## Discussion

### Principal Results

This study used a large health care database comprising 931 sites across the United States to identify changes in use trends for NMB and NMB reversal agents for inpatients before and after health care systems experienced the COVID-19 outbreak. Through multivariable regression analysis, we identified factors related to the patient, procedure, and institution that were associated with NMB reversal choices.

We originally hypothesized that the use of sugammadex for NMB reversal in the post–COVID-19 period would increase given the evidence demonstrating decreased PACU time and, thus, diminished potential exposure to COVID-19. When analyzing changes in the sugammadex use trend for NMB reversal, a slight, transient effect was observed during the post–COVID-19 time points. During EC, a small but statistically significant increase in sugammadex use (compared to neostigmine) was revealed, though this trend was negated by an equivalent decrease during the MC period. However, the association with sugammadex use was small and short-lived, thus arguing that other factors in the complex process of NMB and NMB reversal selection are influencing these decisions. Logistic regression analysis showed that sugammadex use was favored in emergency and urgent admissions compared to elective admissions, and the number of emergency and urgent admissions increased from 42.5% (348,840/820,078) in the BC period to 49.7% (104,123/209,451) in the EC period (Table 1). However, this did not translate to an increase in sugammadex reversal in the counterfactual analysis (model 2b, Figure 4), which largely showed no significant difference in the sugammadex reversal rates of patients being treated in the COVID-19 eras relative to how they would have been treated before COVID-19. It is also important to point out that this study is attempting to identify an association or change in trend, beyond the currently established time-trend, which has observed a strong, steady increase in sugammadex before COVID-19, likely due to increasing evidence of the benefit of sugammadex in avoiding rNMB and in quicker time to reach a train-of-four (TOF) ratio of >0.9 [17,30]. To help account for this, the analysis used data starting in January 2018, which showed a more linear and predictable increase in sugammadex use leading to the start of the COVID-19 pandemic in March 2020. Given the strong association of sugammadex in the yearly time-trend (OR 1.388, 95% CI 1.381-1.396), it is possible that small but significant changes to the NMB reversal trend following COVID-19 are being masked by the existing time-trend.

The counterfactual analysis in this study was intended to identify trends in patient and institutional characteristics that deviated from overall sugammadex reversal patterns captured in the multivariate analysis, which assumes constant effects for all of the covariates. Most of the changes that deviated from the prepandemic trend, as observed from the significantly higher or lower actual or counterfactual ratio in Figure 4, were a reversal of the characteristics that were found to be associated with the early adoption of sugammadex [9]. For example, a lower-than-predicted use of sugammadex in trauma center patients and large hospitals in the peri–COVID-19 time periods may be explained as a renormalization caused by a higher-than-expected adoption of sugammadex in these settings in the initial years after sugammadex approval.

### Limitations

Though this study used robust methodology and a large US database of over 3.5 million inpatient encounters, several limitations must be addressed. The PHD includes patients covered by all payer types from both teaching and nonteaching institutions of various bed sizes. However, it is not representative of geographic location, with the South region more heavily weighted, which could limit the generalizability of the results. The PHD did not include information on the depth of NMB block (moderate vs deep), use of quantitative neuromuscular monitoring, or detection of postoperative rNMB, which can impact NMB reversal selection. The PHD also did not provide individual hospital data on census or capacity limitations. Methods were proposed to account for controlling for the variability in experience between regions, sites, and time periods relative to this capacity. However, there was still a possibility that the impact of a burden on each hospital was either captured incorrectly in general or relative to the BC experience, though the burden was likely to be just as well or just as poorly, captured from one site to another. This study does not take into account reporting sites that were not continuously enrolled throughout this study’s period. During this study’s period extending over 4.5 years, practice and policy changes in surgery and anesthesiology were likely to occur that could have influenced the selection of NMB and NMB reversal agents. Additionally, variations in hospital protocols as well as access to reversal agents were not accounted for in this study. These could include external factors on anesthesia practice among institutions, such as adherence to enhanced recovery protocols, quantitative neuromuscular monitoring following surgery, budgetary constraints, and quality initiatives. Certain patient characteristics (eg, American Society of Anesthesiology physical status classification or smoking status) and procedure data (eg, drug dose or duration of procedure) were not available that could impact NMB reversal choice. By the nature of this study and data collection, there is a potential for recording errors. Lastly, the determination of early, mid, and late COVID-19 time periods was largely subjective. In the absence of nationwide, standardized protocols to guide surgical and anesthesia practices in the wake of the COVID-19 outbreak, each institution adapted independently to the pandemic based on available resources and local impacts of the pandemic, which can vary widely over time and location. Despite the limitations of the data source and our limited ability to identify certain details, our study provides an aggregate observation on the effect of the COVID-19 pandemic on NMB reversal practices in non–COVID-19 patients in the United States.

### Comparison With Prior Work

Before COVID-19, rocuronium (with or without succinylcholine) was the predominant NMB used among US inpatient procedures, accounting for approximately 87% (1,432,947/1,644,370) of patient encounters during the BP. Preference for rocuronium over vecuronium continued through the post–COVID-19 time periods, with 93% (583,815/628,197) of encounters using rocuronium (with or without succinylcholine) in LC. These findings were consistent with prior studies on NMB use among US inpatients. Bash et al [9] demonstrated a trend in preference for rocuronium over vecuronium (with or without succinylcholine) from 2014 to 2019 among US inpatients, increasing from 84.3% in 2014 to 90.7% by the first half of 2019. This trend was even more pronounced among US outpatients, with rocuronium (with or without succinylcholine) accounting for over 96% of NMB use during the first half of 2019 [8].

Database studies revealed trends in NMB reversal favoring active over spontaneous (or no pharmacologic agent) reversal. Among US inpatients, the percentage of encounters using spontaneous reversal gradually decreased from 36.5% in 2014 to 34.3% in 2016 [9]. This decreasing trend accelerated in 2016 (with the approval of sugammadex) and reached 27.6% by the first half of 2019. This current study demonstrated that a decreasing trend in the use of spontaneous reversal continued through the COVID-19 time periods. Logistic regression estimates did not reveal any significant association, or change in the trend, between active versus spontaneous reversal during the EC and MC time periods. During LC, a small but significant association was observed showing a decrease in the rate of active reversal change (effective change in OR of time trend from 1.129 to 1.115). Analyses revealed several patient, procedural, and institutional factors with significant associations with the choice of reversal approach. The most pronounced association identified was related to admission type, with emergency, trauma center, and urgent admissions strongly favoring the use of spontaneous reversal compared to elective procedures. The percentage of elective admissions decreased substantially from 55% (451,190/820,078) in BC to 47.1% (98,637/209,451) in EC, and only partially returned during the MC and LC periods. This was likely the result of a nationwide response to postpone nonessential, elective surgeries as a means to limit COVID-19 exposure in hospitals and focus health care resources on managing the pandemic [14,15].

Contrary to our hypothesis, this study suggests that the impact of COVID-19 on NMB reversal selection was generally limited during the post–COVID-19 era throughout the United States. Though the change in trend for sugammadex use was small and transient in the post–COVID-19 era, the steady trend of increasing sugammadex use over neostigmine that started before COVID-19 and continued in the post–COVID-19 era eclipsed the small transient effects of the pandemic. This trend may be attributed to evidence demonstrating certain benefits of sugammadex over neostigmine, including diminished reversal time, more rapid discharge from the PACU, and a lower incidence of rNMB [17,30-32]. However, the lack of an acceleration of sugammadex use during the post–COVID-19 periods may be attributed to several factors, including clinical inertia or a lack of evidence related to the potential reduction in viral exposure associated with quicker NMB reversal time (and earlier extubation in the operating room). Educational programs can help to ensure current standards of care are attained and maintained in the postoperative setting.

Nonetheless, neostigmine remains a reasonable alternative for NMB reversal in certain patients with minimal blockade. Recent guidelines from the American Society of Anesthesiologists and the European Society of Anesthesiology and Intensive Care (both guidelines released after the date of final data collection of this study) confirm neostigmine’s place in therapy and offer recommendations on the appropriate use of this agent in NMB reversal when accompanied with quantitative neuromuscular monitoring [33,34]. American Society of Anesthesiologists recommends quantitative neuromuscular monitoring for all patients and prefers sugammadex over neostigmine at deep, moderate, and shallow depths (ie, TOF ratio <0.4) of NMB induced by rocuronium or vecuronium [33]. Yet, neostigmine is indicated as a reasonable alternative for patients with minimal blockade (ie, TOF ratio = 0.4 to <0.9) [31]. Similarly, the European Society of Anesthesiology and Intensive Care recommends sugammadex for deep, moderate, and shallow NMB (TOF ratio <0.4) induced by rocuronium or vecuronium, while neostigmine can be considered following advanced spontaneous recovery (ie, TOF ratio >0.2) [34]. Future research using databases that collect TOF information would be instrumental in understanding the impact of these guidelines on current trends and outcomes in NMB reversal selection.

### Conclusions

This large, retrospective database study analyzed over 3.5 million inpatient encounters throughout the United States to identify changes in NMB use and reversal trends during the COVID-19 pandemic. We hypothesized that sugammadex use for NMB reversal would accelerate during the post–COVID-19 eras as a means to reduce PACU or operating room time and, subsequently, the risk of COVID-19 exposure. However, our findings demonstrated only a slight, transient acceleration of sugammadex use during the EC that was largely negated with time. This study did not attempt to investigate the reasons for the lack of change in the existing trend in the use of NMB reversal agents. Additional research is needed to better understand how pandemic-related practice changes have affected long-term NMB and reversal selection based on patient, procedural, or institutional factors, and potentially recognize patient subpopulations that experienced greater changes in anesthesia practice during this period.

## Appendix

Multimedia Appendix 1 Tables showing patient attrition, patient and institution characteristics by COVID era and NMB reversal mechanism. NMB: neuromuscular blockade.

## Abbreviations

BC: before COVID-19 era
BP: baseline period
EC: early COVID-19 era
LC: late COVID-19 era
MC: middle COVID-19 era
NMB: neuromuscular blockade
OR: odds ratio
PACU: postanesthesia care unit
PHD: PINC AI Healthcare Database
rNMB: residual neuromuscular blockade
TOF: train-of-four
