# Supplementary material for: Factors Influencing Neuromuscular Blockade Reversal Choice in the United States Before and During the COVID-19 Pandemic: Retrospective Longitudinal Analysis
Source: JMIR Perioper Med. 2024 Jul 22;7:e52278. doi: 10.2196/52278 (PMC11301116; doi:10.2196/52278)
Supplement: Multimedia Appendix 1 [file periop_v7i1e52278_app1.docx]

**Multimedia Appendix 1. Tables showing patient attrition, patient and institution characteristics by COVID era and NMB reversal mechanism. NMB: neuromuscular blockade.**

**Table S1.** Patient attrition.

| **Criteria** | **Total 3/1/2017 - 12/31/2021** | | **Baseline Period 3/1/2017 - 2/29/2019 (N, %)** | | **Before COVID era 3/1/2019 - 2/29/2020 (N, %)** | | **Early COVID era 4/1/2020 - 7/31/2020 (N, %)** | | **Mid-COVID era 8/1/2020 - 12/31/2020 (N, %)** | | **Late COVID era** **1/1/2021 - 12/31/2021 (N, %)** | |
| --- | --- | --- | --- | --- | --- | --- | --- | --- | --- | --- | --- | --- |
|  | **Patients** | **Encounters** | **Patients** | **Encounters** | **Patients** | **Encounters** | **Patients** | **Encounters** | **Patients** | **Encounters** | **Patients** | **Encounters** |
| Inpatient encounter within the study time-period | 26,934,654 (100%) | 39,386,788 (100%) | 13,413,127 (100%) | 18,049,636 (100%) | 7,038,453 (100%) | 8,852,632 (100%) | 2,081,586 (100%) | 2,370,533 (100%) | 2,764,961 (100%) | 3,201,071 (100%) | 5,625,787 (100%) | 6,912,916 (100%) |
| Encounters >= 18 years of age | 22,054,639 (81.9%) | 34,117,698 (86.6%) | 11,129,973 (83%) | 15,605,373 (86.5%) | 5,934,418 (84.3%) | 7,687,042 (86.8%) | 1,765,975 (84.8%) | 2,046,800 (86.3%) | 2,366,298 (85.6%) | 2,789,890 (87.2%) | 4,744,458 (84.3%) | 5,988,593 (86.6%) |
| Encounters with received rocuronium or vecuronium NMB | 4,413,609 (20%) | 4,913,564 (14.4%) | 2,001,907 (18%) | 2,165,293 (13.9%) | 1,043,824 (17.6%) | 1,104,310 (14.4%) | 296,298 (16.8%) | 304,776 (14.9%) | 417,521 (17.6%) | 431,139 (15.5%) | 862,058 (18.2%) | 908,046 (15.2%) |
| Exclude patients with myasthenia gravis | 4,407,362 (99.9%) | 4,906,327 (99.9%) | 1,999,270 (99.9%) | 2,162,384 (99.9%) | 1,042,268 (99.9%) | 1,102,641 (99.8%) | 295,854 (99.9%) | 304,319 (99.9%) | 416,828 (99.8%) | 430,420 (99.8%) | 860,672 (99.8%) | 906,563 (99.8%) |
| Exclude patients who have ever received pyridostigmine therapy | 4,405,197 (100%) | 4,903,707 (99.9%) | 1,998,261 (99.9%) | 2,161,237 (99.9%) | 1,041,750 (100%) | 1,102,064 (99.9%) | 295,697 (99.9%) | 304,150 (99.9%) | 416,613 (99.9%) | 430,194 (99.9%) | 860,221 (99.9%) | 906,062 (99.9%) |
| Exclude patients with renal failure | 3,656,362 (83%) | 4,017,646 (81.9%) | 1,669,152 (83.5%) | 1,790,224 (82.8%) | 857,044 (82.3%) | 901,203 (81.8%) | 237,381 (80.3%) | 243,445 (80%) | 337,828 (81.1%) | 347,775 (80.8%) | 701,559 (81.6%) | 734,999 (81.1%) |
| Exclude encounters with sugammadex and neostigmine | 3,597,274 (98.4%) | 3,944,927 (98.2%) | 1,647,646 (98.7%) | 1,764,986 (98.6%) | 841,283 (98.2%) | 883,525 (98%) | 232,451 (97.9%) | 238,180 (97.8%) | 330,775 (97.9%) | 340,185 (97.8%) | 686,420 (97.8%) | 718,051 (97.7%) |
| Exclude encounters with obstetrical procedures | 3,564,020 (99.1%) | 3,910,725 (99.1%) | 1,632,918 (99.1%) | 1,750,017 (99.2%) | 833,714 (99.1%) | 875,885 (99.1%) | 230,294 (99.1%) | 236,006 (99.1%) | 327,921 (99.1%) | 337,307 (99.2%) | 679,958 (99.1%) | 711,510 (99.1%) |
| Exclude encounters missing ICD procedure code | 3,551,781 (99.7%) | 3,895,730 (99.6%) | 1,626,988 (99.6%) | 1,743,138 (99.6%) | 830,921 (99.7%) | 872,722 (99.6%) | 229,364 (99.6%) | 235,006 (99.6%) | 326,689 (99.6%) | 335,962 (99.6%) | 677,680 (99.7%) | 708,902 (99.6%) |
| Exclude miscellaneous ICD Procedure encounters | 3,348,406 (94.3%) | 3,668,481 (94.2%) | 1,551,877 (95.4%) | 1,661,085 (95.3%) | 788,441 (94.9%) | 827,404 (94.8%) | 209,963 (91.5%) | 215,070 (91.5%) | 303,058 (92.8%) | 311,575 (92.7%) | 624,676 (92.2%) | 653,347 (92.2%) |
| Exclude encounters with unavailable admission type | 3,318,902 (99.1%) | 3,635,111 (99.1%) | 1,536,869 (99%) | 1,644,616 (99 %) | 781,734 (99.1%) | 820,229 (99.1%) | 208,263 (99.2%) | 213,307 (99.2%) | 300,850 (99.3%) | 309,261 (99.3%) | 619,378 (99.2%) | 647,698 (99.1%) |
| Exclude encounters with COVID-19 positive in same or prior month | 3,289,747 (99.1%) | 3,602,887 (99.1%) | 1,536,650 (100%) | 1,644,370 (100%) | 781,602 (100%) | 820,078 (100%) | 204,498 (98.2%) | 209,451 (98.2%) | 292,683 (97.3%) | 300,791 (97.3%) | 600,872 (97%) | 628,197 (97%) |

**Table S2.** Institution characteristics.

|  | **BP**^a^ | **BC**^b^ | **EC**^c^ | **MC**^d^ | **LC**^e^ |
| --- | --- | --- | --- | --- | --- |
|  | **(N=1,644,370)** | **(N=820,078)** | **(N=209,451)** | **(N=300,791)** | **(N=628,197)** |
| **Teaching (n, %)** |  |  |  |  |  |
| No | 759,302 (46.2%) | 356,992 (43.5%) | 93,041 (44.4%) | 132,171 (43.9%) | 269,935 (43%) |
| Yes | 885,068 (53.8%) | 463,086 (56.5%) | 116,410 (55.6%) | 168,620 (56.1%) | 358,262 (57%) |
| **Urban, rural (n, %)** |  |  |  |  |  |
| Rural | 159,170 (9.7%) | 76,665 (9.3%) | 21,423 (10.2%) | 29,697 (9.9%) | 56,785 (9%) |
| Urban | 1,485,200 (90.3%) | 743,413 (90.7%) | 188,028 (89.8%) | 271,094 (90.1%) | 571,412 (91%) |
| **Bedsize (n, %)** |  |  |  |  |  |
| 000-099 | 67,860 (4.1%) | 32,327 (3.9%) | 8,642 (4.1%) | 13,153 (4.4%) | 24,946 (4%) |
| 100-199 | 191,053 (11.6%) | 95,010 (11.6%) | 23,911 (11.4%) | 34,652 (11.5%) | 68,330 (10.9%) |
| 200-299 | 236,893 (14.4%) | 117,934 (14.4%) | 28,091 (13.4%) | 40,117 (13.3%) | 81,829 (13%) |
| 300-399 | 257,752 (15.7%) | 131,399 (16%) | 33,535 (16%) | 46,893 (15.6%) | 98,798 (15.7%) |
| 400-499 | 203,271 (12.4%) | 103,692 (12.6%) | 27,863 (13.3%) | 39,719 (13.2%) | 78,720 (12.5%) |
| 500+ | 687,541 (41.8%) | 339,716 (41.4%) | 87,409 (41.7%) | 126,257 (42%) | 275,574 (43.9%) |
| **Census region** |  |  |  |  |  |
| Midwest | 366,328 (22.3%) | 190,054 (23.2%) | 48,190 (23%) | 70,742 (23.5%) | 152,961 (24.3%) |
| Northeast | 233,311 (14.2%) | 119,684 (14.6%) | 25,221 (12%) | 42,165 (14%) | 92,657 (14.7%) |
| South | 807,845 (49.1%) | 395,055 (48.2%) | 104,029 (49.7%) | 144,926 (48.2%) | 284,318 (45.3%) |
| West | 236,886 (14.4%) | 115,285 (14.1%) | 32,011 (15.3%) | 42,958 (14.3%) | 98,261 (15.6%) |
| **Institution experience with sugammadex** |  |  |  |  |  |
| 0 - <1 year | 364,936 (22.2%) | 37,314 (4.6%) | 3,335 (1.6%) | 2,773 (0.9%) | 4,508 (0.7%) |
| 1 - <2 years | 580,946 (35.3%) | 87,123 (10.6%) | 11,956 (5.7%) | 16,899 (5.6%) | 13,713 (2.2%) |
| >=2 years | 300,943 (18.3%) | 598,298 (73%) | 181,058 (86.4%) | 262,469 (87.3%) | 576,456 (91.8%) |
| Institutions with <3 years of data | 203,468 (12.4%) | 77,565 (9.5%) | 10,291 (4.9%) | 15,073 (5%) | 28,992 (4.6%) |

^a^BP: baseline period 3/1/2017 - 2/29/2019.

^b^BC: before COVID-19 era 3/1/2019 - 2/29/2020.

^c^EC: early COVID-19 era 4/1/2020 - 7/31/2020.

^d^MC: mid COVID-19 era 8/1/2020 - 12/31/2020.

^e^LC: late COVID-19 era 1/1/2021 - 12/31/2021.

**Table S3.** NMB use by encounters over time periods.

|  | **Total 3/1/2017 - 12/31/2021** | | **BP** | | **BC** | | **EC** | | **MC** | | **LC** | |
| --- | --- | --- | --- | --- | --- | --- | --- | --- | --- | --- | --- | --- |
|  | N | % | N | % | N | % | N | % | N | % | N | % |
| Encounters in cohort | 3,602,887 | 100 | 1,644,370 | 100 | 820,078 | 100 | 209,451 | 100 | 300,791 | 100 | 628,197 | 100 |
| 1. Rocuronium or vecuronium without succinylcholine | 3,379,362 | 93.8 | 1,545,370 | 94 | 770,455 | 93.9 | 193,397 | 92.3 | 279,578 | 92.9 | 590,562 | 94 |
| 1a. Rocuronium only | 3,066,301 | 90.7 | 1,368,270 | 88.5 | 707,534 | 91.8 | 178,431 | 92.3 | 259,729 | 92.9 | 552,337 | 93.5 |
| 1b. Vecuronium only | 200,999 | 5.9 | 115,668 | 7.5 | 39,619 | 5.1 | 9,408 | 4.9 | 12,505 | 4.5 | 23,799 | 4 |
| 1c. Rocuronium and vecuronium only | 112,062 | 3.3 | 61,432 | 4 | 23,302 | 3 | 5,558 | 2.9 | 7,344 | 2.6 | 14,426 | 2.4 |
| 2. Rocuronium or vecuronium with succinylcholine | 189,677 | 5.3 | 79,744 | 4.8 | 42,505 | 5.2 | 14,521 | 6.9 | 19,215 | 6.4 | 33,692 | 5.4 |
| 2a. Rocuronium and succinylcholine | 163,350 | 86.1 | 64,677 | 81.1 | 36,904 | 86.8 | 12,714 | 87.6 | 17,577 | 91.5 | 31,478 | 93.4 |
| 2b. Vecuronium and succinylcholine | 17,803 | 9.4 | 11,221 | 14.1 | 3,336 | 7.8 | 1,196 | 8.2 | 838 | 4.4 | 1,212 | 3.6 |
| 2c. Rocuronium and vecuronium and succinylcholine | 8,524 | 4.5 | 3,846 | 4.8 | 2,265 | 5.3 | 611 | 4.2 | 800 | 4.2 | 1,002 | 3 |

^a^BP: baseline period 3/1/2017 - 2/29/2019.

^b^BC: before COVID-19 era 3/1/2019 - 2/29/2020.

^c^EC: early COVID-19 era 4/1/2020 - 7/31/2020.

^d^MC: mid COVID-19 era 8/1/2020 - 12/31/2020.

^e^LC: late COVID-19 era 1/1/2021 - 12/31/2021.

**Table S4.** Patient characteristics (reversal with sugammadex).

| **Patient characteristics** | | **BP**^a^ | **BC**^b^ | **EC**^c^ | **MC**^d^ | **LC**^e^ |
| --- | --- | --- | --- | --- | --- | --- |
|  |  | **(N=417,266)** | **(N=311,227)** | **(N=92,709)** | **(N=138,148)** | **(N=321,268)** |
| **Age^f^ (years)** | | | | | | |
|  | Mean (SD) | 58.9 (16.37) | 59.2 (16.43) | 58.7 (16.83) | 58.8 (16.66) | 59.0 (16.95) |
|  | Min, Max | 18 , 89 | 18 , 89 | 18 , 89 | 18 , 89 | 18 , 89 |
|  | Median (P25, P75) | 61 (48 , 71) | 61 (48 , 71) | 61 (47 , 71) | 61 (48 , 71) | 61 (47 , 72) |
| **Age categorial^f^ (y), n (%)** | | | | | | |
|  | 18-30 | 27,410 (6.6) | 19,946 (6.4) | 6,802 (7.3) | 9,470 (6.9) | 22,470 (7) |
|  | 31-40 | 37,242 (8.9) | 28,137 (9) | 8,815 (9.5) | 13,298 (9.6) | 31,299 (9.7) |
|  | 41-50 | 54,943 (13.2) | 40,025 (12.9) | 11,988 (12.9) | 18,004 (13) | 41,393 (12.9) |
|  | 51-60 | 86,003 (20.6) | 61,400 (19.7) | 17,865 (19.3) | 26,537 (19.2) | 59,066 (18.4) |
|  | 61-70 | 104,819 (25.1) | 77,750 (25) | 22,563 (24.3) | 33,951 (24.6) | 76,641 (23.9) |
|  | 71-80 | 73,205 (17.5) | 57,937 (18.6) | 16,915 (18.2) | 25,485 (18.4) | 61,574 (19.2) |
|  | >80 | 33,644 (8.1) | 26,032 (8.4) | 7,761 (8.4) | 11,403 (8.3) | 28,825 (9) |
| Sex Female^f^, n (%) | | 231,852 (55.6) | 171,495 (55.1) | 49,534 (53.4) | 75,740 (54.8) | 176,806 (55) |
| **Race^f^, n (%)** | | | | | | |
|  | Asian | 5,988 (1.4) | 4,935 (1.6) | 1,683 (1.8) | 2,451 (1.8) | 7,087 (2.2) |
|  | Black | 39,354 (9.4) | 32,025 (10.3) | 9,602 (10.4) | 15,537 (11.2) | 36,433 (11.3) |
|  | White | 341,036 (81.7) | 245,880 (79) | 73,122 (78.9) | 108,090 (78.2) | 247,680 (77.1) |
| Hispanic ethnicity^f^, n (%) | | 40,582 (9.7) | 27,337 (8.8) | 7,893 (8.5) | 12,742 (9.2) | 33,781 (10.5) |
| **Insurance^f,h^, n (%)** | | | | | | |
|  | Commercial | 164,166 (39.3) | 118,921 (38.2) | 34,644 (37.4) | 52,568 (38.1) | 117,010 (36.4) |
|  | Government | 193,577 (46.4) | 147,762 (47.5) | 43,320 (46.7) | 64,269 (46.5) | 151,277 (47.1) |
|  | Low-income | 54,751 (13.1) | 41,021 (13.2) | 13,651 (14.7) | 19,805 (14.3) | 48,799 (15.2) |
| Comorbidites>=1^f^, n (%) | | 334,071 (80.1) | 257,006 (82.6) | 78,053 (84.2) | 115,973 (83.9) | 273,297 (85.1) |
| **Comorbidities^i^, n (%)** | | | | | | |
|  | Cardiac arrhythmias^f^ | 61,803 (14.8) | 50,525 (16.2) | 15,746 (17) | 23,187 (16.8) | 56,746 (17.7) |
|  | Chronic pulmonary disease^f^ | 79,593 (19.1) | 62,178 (20) | 18,827 (20.3) | 27,989 (20.3) | 65,196 (20.3) |
|  | Congestive heart failure^f^ | 24,236 (5.8) | 21,268 (6.8) | 6,874 (7.4) | 9,972 (7.2) | 25,004 (7.8) |
|  | Depression^f^ | 56,516 (13.5) | 45,197 (14.5) | 13,557 (14.6) | 21,106 (15.3) | 48,547 (15.1) |
|  | Diabetes (complicated)^f^ | 30,456 (7.3) | 26,309 (8.5) | 8,915 (9.6) | 12,537 (9.1) | 30,915 (9.6) |
|  | Diabetes (uncomplicated)^f^ | 56,125 (13.5) | 41,326 (13.3) | 11,752 (12.7) | 18,077 (13.1) | 41,895 (13) |
|  | Fluid or electrolyte disorders^f^ | 71,984 (17.3) | 58,585 (18.8) | 20,900 (22.5) | 28,864 (20.9) | 69,532 (21.6) |
|  | Hypothyroidism^f^ | 51,851 (12.4) | 40,315 (13) | 11,694 (12.6) | 17,632 (12.8) | 41,473 (12.9) |
|  | Obesity or overweight^f^ | 103,131 (24.7) | 83,391 (26.8) | 24,938 (26.9) | 39,329 (28.5) | 91,228 (28.4) |
|  | Other neurological disorders^f^ | 25,991 (6.2) | 20,965 (6.7) | 7,086 (7.6) | 10,221 (7.4) | 24,549 (7.6) |
|  | Peripheral vascular disorders^f^ | 30,351 (7.3) | 24,991 (8) | 7,828 (8.4) | 11,853 (8.6) | 28,880 (9) |
|  | Sleep apnea^f^ | 37,867 (9.1) | 31,666 (10.2) | 9,032 (9.7) | 14,445 (10.5) | 34,568 (10.8) |
|  | Solid tumor without metastasis^f^ | 55,987 (13.4) | 42,903 (13.8) | 13,658 (14.7) | 19,124 (13.8) | 46,745 (14.6) |
| COVID-19 not present^f,j^, n (%) | | 417,209 (100) | 311,138 (100) | 92,609 (99.9) | 137,466 (99.5) | 307,668 (95.8) |
| **Admission type^f^, n (%)** | | | | | | |
|  | Elective | 239,219 (57.3) | 173,550 (55.8) | 44,478 (48) | 72,961 (52.8) | 159,526 (49.7) |
|  | Emergency or urgent | 166,710 (40) | 129,178 (41.5) | 45,056 (48.6) | 61,296 (44.4) | 152,228 (47.4) |
|  | Trauma center | 11,337 (2.7) | 8,499 (2.7) | 3,175 (3.4) | 3,891 (2.8) | 9,514 (3) |

^a^BP: baseline period 3/1/2017 - 2/29/2019.

^b^BC: before COVID-19 era 3/1/2019 - 2/29/2020.

^c^EC: early COVID-19 era 4/1/2020 - 7/31/2020.

^d^MC: mid COVID-19 era 8/1/2020 - 12/31/2020.

^e^LC: late COVID-19 era 1/1/2021 - 12/31/2021.

^f^Statistically significant at the *P*<.05 level.

^g^P25: 25^th^ percentile; P75: 75^th^ percentile.

^h^Commercial category includes managed care, and workers compensation, and self-pay. Government category includes Medicare and other government insurance type. Low-income category includes Medicaid, charity, and indigent.

^i^Most frequently observed Elixhauser comorbidities shown

^j^No history of COVID-19 within 2 months of encounter.

**Table S5.** Patient characteristics (reversal with neostigmine).

| **Patient characteristics** | | **BP**^a^ | **BC**^b^ | **EC**^c^ | **MC**^d^ | **LC**^e^ |
| --- | --- | --- | --- | --- | --- | --- |
|  |  | **(N=775,266)** | **(N=307,727)** | **(N=67,321)** | **(N=94,181)** | **(N=167,075)** |
| **Age^f^ (years)** | | | | | | |
|  | Mean (SD) | 57.8 (16.49) | 58.2 (16.56) | 57.7 (16.98) | 57.8 (16.81) | 57.4 (17.15) |
|  | Min, Max | 18 , 89 | 18 , 89 | 18 , 89 | 18 , 89 | 18 , 89 |
|  | Median (P25, P75) | 60 (47 , 70) | 60 (47 , 71) | 60 (46 , 71) | 60 (46 , 71) | 60 (45 , 70) |
| **Age categorial^f^ (y), n (%)** | | | | | | |
|  | 18-30 | 57,079 (7.4) | 22,364 (7.3) | 5,531 (8.2) | 7,377 (7.8) | 14,038 (8.4) |
|  | 31-40 | 75,856 (9.8) | 29,845 (9.7) | 6,840 (10.2) | 9,557 (10.1) | 18,270 (10.9) |
|  | 41-50 | 108,173 (14) | 42,254 (13.7) | 9,154 (13.6) | 12,973 (13.8) | 23,224 (13.9) |
|  | 51-60 | 158,746 (20.5) | 59,872 (19.5) | 12,901 (19.2) | 18,044 (19.2) | 30,890 (18.5) |
|  | 61-70 | 190,538 (24.6) | 75,468 (24.5) | 16,004 (23.8) | 22,675 (24.1) | 38,960 (23.3) |
|  | 71-80 | 130,619 (16.8) | 55,691 (18.1) | 11,931 (17.7) | 16,852 (17.9) | 29,316 (17.5) |
|  | >80 | 54,255 (7) | 22,233 (7.2) | 4,960 (7.4) | 6,703 (7.1) | 12,377 (7.4) |
| Sex Female^f^, n (%) | | 441,284 (56.9) | 173,922 (56.5) | 36,704 (54.5) | 52,430 (55.7) | 93,193 (55.8) |
| **Race^f^, n (%)** | | | | | | |
|  | Asian | 11,328 (1.5) | 4,769 (1.5) | 1,178 (1.7) | 1,723 (1.8) | 3,383 (2) |
|  | Black | 89,998 (11.6) | 35,168 (11.4) | 8,245 (12.2) | 11,835 (12.6) | 22,274 (13.3) |
|  | White | 591,177 (76.3) | 232,842 (75.7) | 51,393 (76.3) | 71,210 (75.6) | 123,883 (74.1) |
| Hispanic ethnicity^f^, n (%) | | 62,290 (8) | 28,993 (9.4) | 6,289 (9.3) | 8,933 (9.5) | 19,997 (12) |
| **Insurance^f,h^, n (%)** | | | | | | |
|  | Commercial | 329,403 (42.5) | 125,807 (40.9) | 27,538 (40.9) | 37,946 (40.3) | 65,977 (39.5) |
|  | Government | 340,107 (43.9) | 137,824 (44.8) | 29,544 (43.9) | 41,364 (43.9) | 71,724 (42.9) |
|  | Low-income | 98,142 (12.7) | 39,788 (12.9) | 9,207 (13.7) | 13,295 (14.1) | 26,068 (15.6) |
| Comorbidites>=1^f^, n (%) | | 608,704 (78.5) | 246,033 (80) | 54,391 (80.8) | 76,277 (81) | 137,412 (82.2) |
| **Comorbidities^i^, n (%)** | | | | | | |
|  | Cardiac arrhythmias^f^ | 107,136 (13.8) | 44,907 (14.6) | 10,349 (15.4) | 14,415 (15.3) | 26,612 (15.9) |
|  | Chronic pulmonary disease^f^ | 134,175 (17.3) | 53,963 (17.5) | 11,662 (17.3) | 16,594 (17.6) | 29,738 (17.8) |
|  | Congestive heart failure^f^ | 38,739 (5) | 16,905 (5.5) | 4,047 (6) | 5,586 (5.9) | 10,892 (6.5) |
|  | Depression^f^ | 100,578 (13) | 42,370 (13.8) | 9,047 (13.4) | 13,174 (14) | 23,594 (14.1) |
|  | Diabetes (complicated)^f^ | 52,392 (6.8) | 23,306 (7.6) | 5,635 (8.4) | 7,838 (8.3) | 14,789 (8.9) |
|  | Diabetes (uncomplicated)^f^ | 103,974 (13.4) | 40,040 (13) | 8,110 (12) | 11,650 (12.4) | 20,422 (12.2) |
|  | Fluid or electrolyte disorders^f^ | 126,067 (16.3) | 53,048 (17.2) | 13,935 (20.7) | 17,892 (19) | 32,841 (19.7) |
|  | Hypothyroidism^f^ | 93,307 (12) | 38,249 (12.4) | 8,006 (11.9) | 11,419 (12.1) | 19,776 (11.8) |
|  | Obesity or overweight^f^ | 191,901 (24.8) | 79,007 (25.7) | 17,120 (25.4) | 25,163 (26.7) | 46,092 (27.6) |
|  | Other neurological disorders^f^ | 40,904 (5.3) | 17,297 (5.6) | 4,199 (6.2) | 5,715 (6.1) | 10,194 (6.1) |
|  | Peripheral vascular disorders^f^ | 51,314 (6.6) | 21,674 (7) | 5,081 (7.5) | 7,142 (7.6) | 13,706 (8.2) |
|  | Sleep apnea^f^ | 68,893 (8.9) | 29,692 (9.6) | 5,728 (8.5) | 8,901 (9.5) | 16,235 (9.7) |
|  | Solid tumor without metastasis^f^ | 97,505 (12.6) | 37,796 (12.3) | 8,986 (13.3) | 11,427 (12.1) | 21,563 (12.9) |
| COVID-19 not present^f,j^, n (%) | | 775,173 (100) | 307,655 (100) | 67,239 (99.9) | 93,738 (99.5) | 160,199 (95.9) |
| **Admission type^f^, n (%)** | | | | | | |
|  | Elective | 466,803 (60.2) | 182,677 (59.4) | 34,906 (51.9) | 53,567 (56.9) | 89,430 (53.5) |
|  | Emergency or urgent | 295,303 (38.1) | 119,968 (39) | 30,872 (45.9) | 38,826 (41.2) | 73,656 (44.1) |
|  | Trauma center | 13,160 (1.7) | 5,082 (1.7) | 1,543 (2.3) | 1,788 (1.9) | 3,989 (2.4) |

^a^BP: baseline period 3/1/2017 - 2/29/2019.

^b^BC: before COVID-19 era 3/1/2019 - 2/29/2020.

^c^EC: early COVID-19 era 4/1/2020 - 7/31/2020.

^d^MC: mid COVID-19 era 8/1/2020 - 12/31/2020.

^e^LC: late COVID-19 era 1/1/2021 - 12/31/2021.

^f^Statistically significant at the *P*<.05 level.

^g^P25: 25^th^ percentile; P75: 75^th^ percentile.

^h^Commercial category includes managed care, and workers compensation, and self-pay. Government category includes Medicare and other government insurance type. Low-income category includes Medicaid, charity, and indigent.

^i^Most frequently observed Elixhauser comorbidities shown

^j^No history of COVID-19 within 2 months of encounter.

**Table S6.** Patient characteristics (no active reversal agent).

| **Patient characteristics** | | **BP**^a^ | **BC**^b^ | **EC**^c^ | **MC**^d^ | **LC**^e^ |
| --- | --- | --- | --- | --- | --- | --- |
|  |  | **(N=451,838)** | **(N=201,124)** | **(N=49,421)** | **(N=68,462)** | **(N=139,854)** |
| **Age^f^ (years)** | | | | | | |
|  | Mean (SD) | 59.6 (15.88) | 59.9 (15.83) | 59.2 (16.28) | 59.5 (15.99) | 59.7 (16.15) |
|  | Min, Max | 18 , 89 | 18 , 89 | 18 , 89 | 18 , 89 | 18 , 89 |
|  | Median (P25, P75) | 62 (50 , 71) | 62 (50 , 71) | 61 (49 , 71) | 62 (50 , 71) | 62 (50 , 72) |
| **Age categorial^f^ (y), n (%)** | | | | | | |
|  | 18-30 | 27,644 (6.1) | 11,915 (5.9) | 3,350 (6.8) | 4,281 (6.3) | 8,830 (6.3) |
|  | 31-40 | 34,865 (7.7) | 15,256 (7.6) | 4,184 (8.5) | 5,612 (8.2) | 11,462 (8.2) |
|  | 41-50 | 53,956 (11.9) | 23,391 (11.6) | 5,901 (11.9) | 8,115 (11.9) | 16,251 (11.6) |
|  | 51-60 | 96,012 (21.2) | 41,495 (20.6) | 10,107 (20.5) | 13,868 (20.3) | 27,246 (19.5) |
|  | 61-70 | 121,386 (26.9) | 54,161 (26.9) | 12,737 (25.8) | 18,309 (26.7) | 37,445 (26.8) |
|  | 71-80 | 84,370 (18.7) | 39,749 (19.8) | 9,409 (19) | 13,347 (19.5) | 27,937 (20) |
|  | >80 | 33,605 (7.4) | 15,157 (7.5) | 3,733 (7.6) | 4,930 (7.2) | 10,683 (7.6) |
| Sex Female^f^, n (%) | | 217,774 (48.2) | 94,900 (47.2) | 22,303 (45.1) | 31,723 (46.3) | 63,932 (45.7) |
| **Race^f^, n (%)** | | | | | | |
|  | Asian | 7,889 (1.7) | 3,451 (1.7) | 950 (1.9) | 1,328 (1.9) | 3,477 (2.5) |
|  | Black | 42,965 (9.5) | 19,426 (9.7) | 4,973 (10.1) | 7,049 (10.3) | 16,084 (11.5) |
|  | White | 355,332 (78.6) | 155,334 (77.2) | 38,503 (77.9) | 53,206 (77.7) | 106,211 (75.9) |
| Hispanic ethnicity^f^, n (%) | | 38,311 (8.5) | 17,507 (8.7) | 4,244 (8.6) | 6,178 (9) | 15,107 (10.8) |
| **Insurance^f,h^, n (%)** | | | | | | |
|  | Commercial | 171,872 (38) | 73,244 (36.4) | 17,727 (35.9) | 24,499 (35.8) | 48,553 (34.7) |
|  | Government | 215,705 (47.7) | 97,432 (48.4) | 23,181 (46.9) | 32,419 (47.4) | 66,799 (47.8) |
|  | Low-income | 59,280 (13.1) | 27,700 (13.8) | 7,789 (15.8) | 10,637 (15.5) | 22,343 (16) |
| Comorbidites>=1^f^, n (%) | | 379,136 (83.9) | 173,452 (86.2) | 43,495 (88) | 60,105 (87.8) | 124,367 (88.9) |
| **Comorbidities^i^, n (%)** | | | | | | |
|  | Cardiac arrhythmias^f^ | 109,560 (24.2) | 52,942 (26.3) | 13,706 (27.7) | 18,819 (27.5) | 41,098 (29.4) |
|  | Chronic pulmonary disease^f^ | 90,103 (19.9) | 41,261 (20.5) | 10,319 (20.9) | 14,312 (20.9) | 28,931 (20.7) |
|  | Congestive heart failure^f^ | 51,877 (11.5) | 26,664 (13.3) | 7,197 (14.6) | 9,755 (14.2) | 21,785 (15.6) |
|  | Depression^f^ | 59,141 (13.1) | 27,658 (13.8) | 6,810 (13.8) | 9,970 (14.6) | 19,687 (14.1) |
|  | Diabetes (complicated)^f^ | 48,746 (10.8) | 25,045 (12.5) | 6,680 (13.5) | 9,035 (13.2) | 20,132 (14.4) |
|  | Diabetes (uncomplicated)^f^ | 63,215 (14) | 27,258 (13.6) | 6,414 (13) | 8,865 (12.9) | 18,033 (12.9) |
|  | Fluid or electrolyte disorders^f^ | 127,768 (28.3) | 62,848 (31.2) | 17,651 (35.7) | 23,262 (34) | 50,206 (35.9) |
|  | Hypothyroidism^f^ | 54,971 (12.2) | 25,048 (12.5) | 6,004 (12.1) | 8,486 (12.4) | 17,162 (12.3) |
|  | Obesity or overweight^f^ | 99,389 (22) | 48,290 (24) | 12,224 (24.7) | 17,759 (25.9) | 36,347 (26) |
|  | Other neurological disorders^f^ | 50,639 (11.2) | 25,090 (12.5) | 7,216 (14.6) | 9,575 (14) | 20,628 (14.7) |
|  | Peripheral vascular disorders^f^ | 43,413 (9.6) | 20,843 (10.4) | 5,430 (11) | 7,600 (11.1) | 16,111 (11.5) |
|  | Sleep apnea^f^ | 41,091 (9.1) | 20,347 (10.1) | 4,738 (9.6) | 6,913 (10.1) | 14,376 (10.3) |
|  | Solid tumor without metastasis^f^ | 40,671 (9) | 17,976 (8.9) | 4,658 (9.4) | 6,093 (8.9) | 12,837 (9.2) |
| COVID-19 not present^f,j^, n (%) | | 451,760 (100) | 201,059 (100) | 49,352 (99.9) | 68,092 (99.5) | 133,603 (95.5) |
| **Admission type^f^, n (%)** | | | | | | |
|  | Elective | 226,586 (50.1) | 94,963 (47.2) | 19,253 (39) | 29,951 (43.7) | 58,047 (41.5) |
|  | Emergency or urgent | 212,310 (47) | 99,694 (49.6) | 28,195 (57.1) | 36,059 (52.7) | 76,324 (54.6) |
|  | Trauma center | 12,942 (2.9) | 6,467 (3.2) | 1,973 (4) | 2,452 (3.6) | 5,483 (3.9) |

^a^BP: baseline period 3/1/2017 - 2/29/2019.

^b^BC: before COVID-19 era 3/1/2019 - 2/29/2020.

^c^EC: early COVID-19 era 4/1/2020 - 7/31/2020.

^d^MC: mid COVID-19 era 8/1/2020 - 12/31/2020.

^e^LC: late COVID-19 era 1/1/2021 - 12/31/2021.

^f^Statistically significant at the *P*<.05 level.

^g^P25: 25^th^ percentile; P75: 75^th^ percentile.

^h^Commercial category includes managed care, and workers compensation, and self-pay. Government category includes Medicare and other government insurance type. Low-income category includes Medicaid, charity, and indigent.

^i^Most frequently observed Elixhauser comorbidities shown

^j^No history of COVID-19 within 2 months of encounter.
